# A Meta-Analysis of Randomized Controlled Trials to Compare Long-Term Outcomes of Nissen and Toupet Fundoplication for Gastroesophageal Reflux Disease

**DOI:** 10.1371/journal.pone.0127627

**Published:** 2015-06-29

**Authors:** Zhi-chao Tian, Bin Wang, Cheng-xiang Shan, Wei Zhang, Dao-zhen Jiang, Ming Qiu

**Affiliations:** Department of General Surgery of Changzheng Hospital affiliated to Second Military Medical University, No.415 Fengyang road, Shanghai, 200003 China; University of Florida, UNITED STATES

## Abstract

**Aim:**

In recent years, several studies with large sample sizes and recent follow-up data have been published comparing outcomes between laparoscopic Nissen fundoplication and laparoscopic Toupet fundoplication. It is now timely to be re-evaluated and synthesized long-term efficacy and adverse events of both total and partial posterior fundoplication.

**Materials and Methods:**

Electronic searches for RCTs comparing the outcome after laparoscopic Nissen fundoplication and laparoscopic Toupet fundoplication were performed in the databases of MEDLINE, EMBASE, and the Cochrane Center Register of Controlled Trials. The data of evaluation in positive and adverse results of laparoscopic Nissen fundoplication and laparoscopic Nissen fundoplication were extracted and compared using meta-analysis.

**Results:**

13 RCTs were ultimately identified involving 814 (52.05%) and 750 (47.95%) patients who underwent laparoscopic Nissen fundoplication and laparoscopic Toupet fundoplication, respectively. The operative time, perioperative complications, postoperative satisfaction, recurrence, and the rates of medication adoption or re-operation due to recurrence were not significantly different between two groups. The two types of fundoplication both reinforced the anti-reflux barrier and elevated the lower esophageal sphincter pressure. However, rates of adverse results involving dysphasia, gas-bloat syndrome, inability to belch and re-operation due to severe dysphasia were significantly higher after LNF. In the subgroup analysis of wrap length≤2cm, laparoscopic Nissen fundoplication was associated with a significantly higher incidence of postoperative dysphagia. However, in the subgroup wrap length>2cm, the difference was not statistically significant.

**Conclusion:**

Laparoscopic Toupet fundoplication might be the better surgery approach for gastroesophageal reflux disease with a lower rate of postoperative adverse results and equal effectiveness as Laparoscopic Nissen fundoplication.

## Introduction

The lower esophageal sphincter (LES) plays an important role in the pathogenesis of gastroesophageal reflux disease (GERD) [1]. In order to inhibit LES relaxation, fundoplication is considered to be an essential and important part of antireflux surgery. Laparoscopic Nissen fundoplication (LNF), a total wrap that surrounds the esophagus 360°, is the most commonly used, gold standard technique worldwide for antireflux surgery [2]. However, LNF is associated with a high incidence of postoperative dysphagia and gas-bloat syndrome [3,4]. Laparoscopic Toupet fundoplication (LTF), a 270° partial wrap, was introduced to counteract these side effects. An ongoing discussion has focused on the ideal approach to antireflux surgery, including durable reflux control, as well as minimal postoperative dysphagia and gas-related symptoms.

Many surgeons advocate that the incidence of regurgitation and heartburn are similar in both LNF and LTF, while postoperative dysphagia may have a higher incidence following LNF [5]. Besides fundoplication type, other variables such as the length of the wrap and impaired esophageal peristalsis may also be associated with postoperative dysphagia [6,7]. The original LNF procedure, which purports a 6-cm wrap length, is associated with a higher dysphagia rate [8]. Two studies on the length of the Nissen fundoplication showed that a loose wrap of 1–2 cm was sufficient to suppress reflux and reduce the incidence of postoperative bloating and dysphagia [9,10]. An early study showed that LTF was more effective when esophageal motility (EM) was abnormal (less than 50% peristaltic waveforms) [11]. However, a previous study reported a similar incidence of dysphagia between LTF and LNF 1 year postoperatively [12]. Whether LTF has a benefit on abnormal esophageal peristalsis remains controversial.

Several meta-analyses have been performed comparing outcomes between LNF and LTF until 2011 [13,14]. However, a comprehensive study collecting randomized clinical trials (RCTs) has not been conducted to date. In recent years, several studies with large sample sizes and recent follow-up data have been published comparing long-term efficacy and adverse events of both total and partial fundoplication [7, 15–17]. Therefore, in order to better weigh the potential benefits against the potential side effects, data from these recent trials is now timely to be re-evaluated and synthesized with the existing trials. To address this need, we performed a meta-analysis of RCTs to determine the optimal surgical approach for GERD, providing better reflux control with minimal postoperative complications.

## Methods

### Search strategy

All RCTs in the English language comparing outcomes of LNF with LTF were eligible for the meta-analysis, regardless of publication status (published, unpublished, in press, or in progress).

Electronic searches were performed for relevant reports in the MEDLINE, EMBASE, and the Cochrane Center Register of Controlled Trials databases, until June 2014. We adopted our search strategy using disease-specific terms (e.g. gastroesophageal reflux disease), management-specific terms (e.g. laparoscopic fundoplication), and terms related to surgical procedures (e.g. Nissen, Toupet, partial, and total). The abstracts of all potential articles, references, and related articles were reviewed according to their titles.

**Fig 1:**
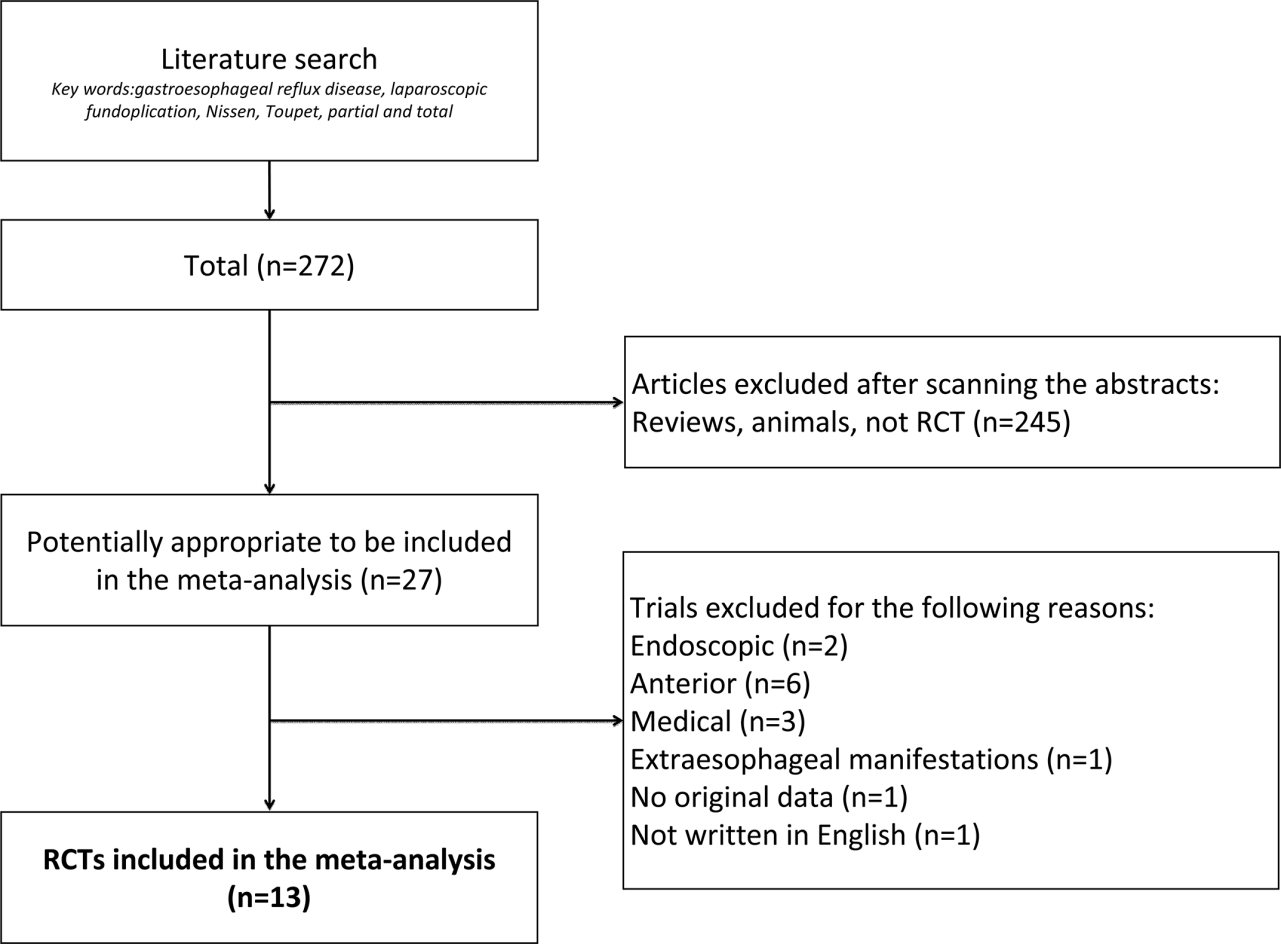
A flow chart showing the details of search strategy and the trials selection process according to the including and excluding criteria.

Each article was independently assessed for eligibility using inclusion and exclusion criteria. Inclusion criteria were as follows: (1) randomized controlled trials (RCTs) comparing efficacy and negative outcomes of laparoscopic fundoplication (LF), including LNF and LTF; and (2) exact and intact dichotomous-type or continuous-type data with standard deviations. Exclusion criteria were as follows: (1) non-randomized controlled trials; (2) trials comparing total and non-posterior partial fundoplication (e.g. total vs. anterior partial fundoplication); and (3) patients younger than 16 years. The literature search was performed independently by two authors (Bin Wang and Zhi-chao Tian). The third author (Ming Qiu) determined whether or not to include the study when controversy occurred. Our procedure for screening and selection is shown in Fig 1.

The criteria for grading methodologic quality of included studies was determined by group discussion. The determination of whether methodologic bias existed was made according to Cochrane criteria guidelines, including selection, performance, detection, attrition, reporting, and other biases [18]. All items assessed were shown in Forest-plot figures.

### Selection criteria

Two investigators (Bin Wang and Zhi-chao Tian) independently extracted and collected data using a standardized data-extraction protocol. Data of baseline patient characteristics were collected, including first author, publication year, sample size, age, sex, BMI, wrap length (cm), and duration of mean follow-up. For repeated publications, we only extracted the most recent data of interesting outcomes. For example, Mickevicius et al. reported their results twice at different periods of the same trial [6, 7]. Therefore, we extracted different outcomes from the corresponding article (e.g. perioperative complications from Mickevicius et al. 2008 [6], and dysphagia from Mickevicius et al. 2013 [7] for long-term outcome evaluation. Otherwise, Mickevicius et al. [6, 7] further reported effectiveness and adverse results according to the length of wrap (1.5 cm vs. 3.0 cm). Therefore, we extracted and showed data from the 1.5 cm group and 3.0 cm group, respectively, in some fields.

### Statistical analysis

Data extracted from included trials were integrated with Review Manager Software version 5.2 (Cochrane Collaborative, Oxford, England). The risk ratio was used for dichotomous-type data and the standard mean difference for continuous-type data. The fixed-effects model was used only if no significant heterogeneity was detected among trials, or if the random-effects model was adopted to ignore heterogeneity. Subgroup analyses were performed to assess the impact of esophageal motility and wrap length on dysphagia, according to esophageal function (subgroup 1: EM both normal; subgroup 2: EM both abnormal; subgroup 3: EM normal before LNF vs. abnormal before LTF) and wrap length (subgroup 1: WL ≤2 cm; subgroup 2: WL >2 cm).

## Results

### Baseline characteristics of included trials

Finally, the literature search identified 13 RCTs [6, 7, 12, 15–17, 19, 20–25] published between June 1997 and July 2013. A total of 814 (52.05%) patients underwent LNF and 750 (47.95%) patients underwent LTF. The LNF group was comprised of 212 (57.92%) males and 207 (58.97%) females in the LTF, respectively. Patient ages ranged from 19 to 82 years. Five trials [7, 16, 20, 24–25] calculated the body mass index (BMI) of patients, which were similar within a range from 26 to 29 kg/m^2^. More preoperative details are shown in Table 1.

**Table 1:** The baseline characteristics of included trials.

Included references	Sample Capcity*	Follow-up (mo)	Sex**	Age(yr) *	Weight(Kg) or BMI(Kg/m^2^) *	Wrap length (cm) *	PPI use (%)*	Duration of symptoms*
**Laws 1997[19]**	23/16	Mean 27.2	19/20	45.5 / 55.5	No detail	Average 2.2 [1.6–3] / -	No detail	No detail
**Chrysos 2003[12]**	14/19	12	18/15	61.7±8.7 / 59.2±11.5	No detail	3–4 / 3–4	No detail	No detail
**Guérin 2007[20]**	77/63	36	86/54	No detail	Median BMI 27/26.6	3 / -	No detail	No detail
**Booth 2008[21]**	64/63	12	84/43	45.3[21–86]/44.2[19–69]	Mean Weight 81.6/80.2	2 / -	92% / 90%	94.5 (7–516) / 95.6 (6–248) mo
**Fibbe 2001[22]**	100/100	24	121/79	56[20–80]	Mean BMI 26.4[18.9–40.4]	2 / -	Average 24 (0.2–180) mo	Median 7 (0.2–50) yr
**Zornig 2002[23]**								
**Strate 2008[24]**								
**Shaw 2010[25]**	50/50	60	60/40	45.2[28–72]/45.6[25–67]	Mean BMI 29.3±5.2/29.2±5.2	1 / 2	No detail	2.36±0.5 / 2.58±0.6
**Mucio 2012[15]**	133/131	180	No detail	No detail	No detail	2.54 (1 inch) / -	No detail	No detail
**Koch 2013[16]**	62/63	12	78/47	50.32[20–76]/51.87[25–81]	Mean BMI 28.18[19.47–41.80]/27.32[19.66–3.86]	- / -	No detail	No detail
**Qin 2013[17]**	215/168	12	194/189	56.3[34–82]	No detail	- / -	No detail	No detail
**Mickevicius 2008[6]**	76/77	60	74/79	49.2±14.4 / 54.8±12.6	Mean BMI 27.7±4.6/29.3±3.2	1.5 *vs*. 3 / 1.5 *vs*. 3	No detail	7.2±7.7 / 7.1±7.6 yr
**Mickevicius 2013[7]**								

LNF: Laparoscopic Nissen fundoplication; LTF: Laparoscopic Toupet fundoplication; Mo: Month; yr: year; BMI: Body mass index; PPI: Proton pump inhibitors.

*Data in these columns are showed as data in LNF group/data in LTF group

**Data are showed as number of males/number of female

### Perioperative characteristics

#### Operative time and hospital stay

Seven trials [6, 12, 15, 17, 19, 21, 25] reported mean operative time (S2 Fig), which ranged from 35.5 [15] to 155 [19] min in the LNF group and 32.5 [15] to 162 [19] min in the LTF group. Operative duration was shorter in the LNF group, but no significant difference was found between groups (LNF vs. LTF, standard mean difference -0.45, 95% CI [-1.00, 0.11], p = 0.11). Four trials reported mean hospital stay in both groups, which ranged from 2.6 [12] to 3.8 [17] days in the LNF group and from 2.5 [19] to 4.2 [17] days in the LTF group.

#### Perioperative complications

Five trials [7, 12, 15, 19, 21] reported perioperative complications (S3 Fig), which occurred in 19 patients with LNF and 29 patients with LTF, and included lacerations of the gastric fundus and spleen, bleeding from the spleen or short gastric blood vessels, and pneumothorax. Although the incidence of perioperative complications was slightly lower in the LNF compared with LTF group, this trend was not found to be statistically significant (6.13% vs. 9.48%, RR 0.67, 95% CI [0.39, 1.14], p = 0.14).

### Postoperative satisfaction

Four trials [12, 20, 24, 25] evaluated patient satisfaction during the follow-up period (S4 Fig), which were categorized as excellent, good, unchanged, or worse. Overall satisfaction rates were remarkably high after both LNF and LTF, without a significant difference between groups (89.30% LNF vs. 85.38% LTF, RR 1.05, 95% CI [0.97, 1.13], p = 0.22). The mean general gastrointestinal quality of life index score in the LNF and LTF group was 96.3 ± 16.6/93.7 ± 21.2 preoperatively and 119.8 ± 15.71/115.2 ± 15.96 postoperatively [16]. Healthy individuals scored, on average, 122.6 ± 8.5 points.

### Postoperative symptoms

#### Dysphagia

**Fig 2:**
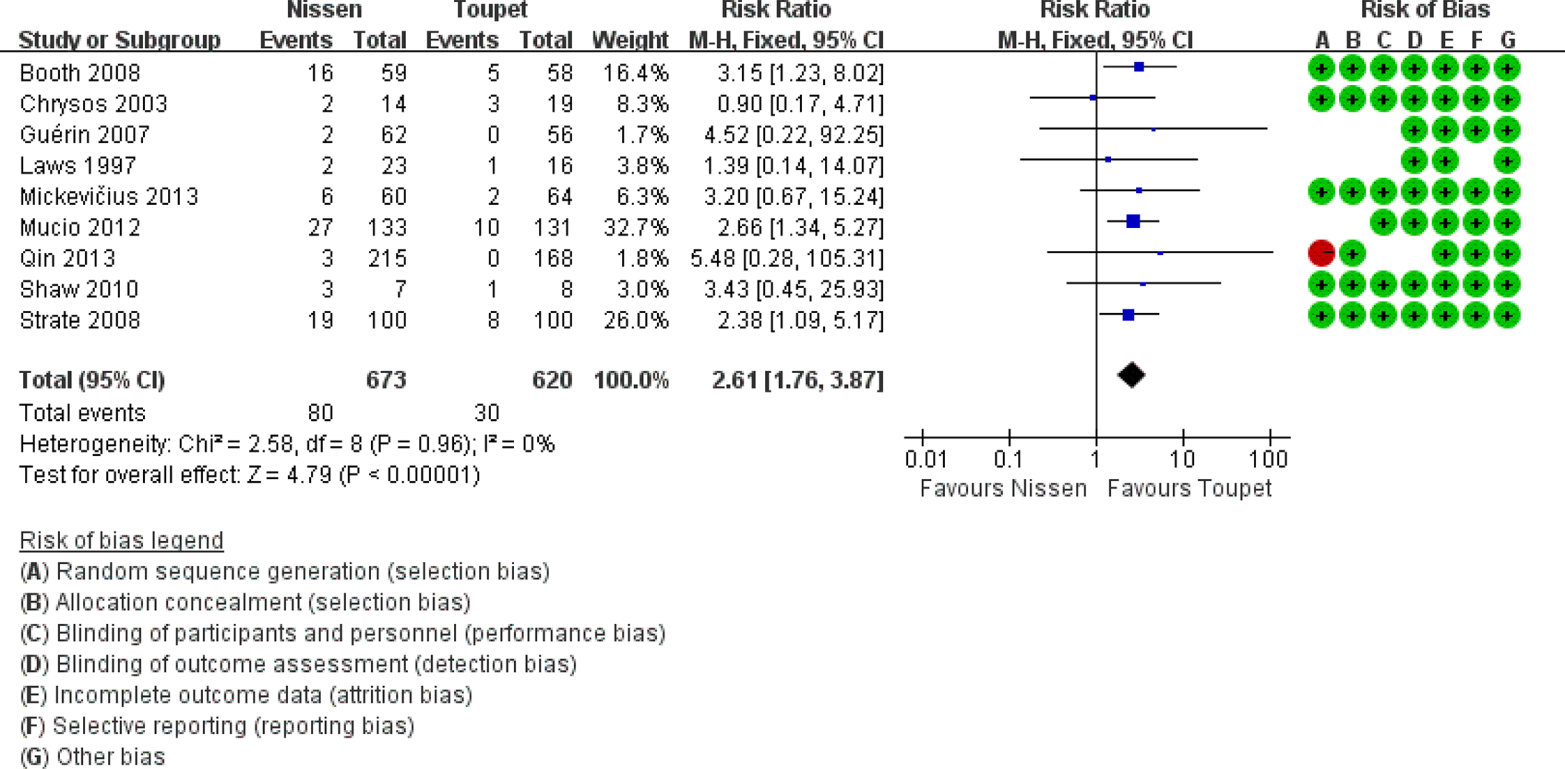
Overall rates of dysphagia after LNF and LTF.

Nine trials [7, 12, 15, 17, 19, 20–21, 24–25] reported postoperative dysphagia (Fig 2), which occurred in 80/637 (12.56%) and 30/620 (4.84%) of patients in the LNF and LTF groups, respectively, and was significantly higher after LNF compared with LTF (RR 2.61, 95% CI [1.76, 3.87], p <0.01). Two trials [7,12] evaluated dysphagia severity. Even though the incidence of moderate-to-severe dysphagia failed to show a significant difference between groups (S5 Fig), it was clear that LNF was associated with moderate-to-severe dysphagia (8.86% vs. 3.85%, RR 2.28, 95% CI [0.63, 8.30], p = 0.21). Additionally, 14/100 (14.00%) patients after LNF and 5/100 (5.00%) after LTF had severe dysphagia, requiring endoscopic bougie dilatation [24].

**Fig 3:**
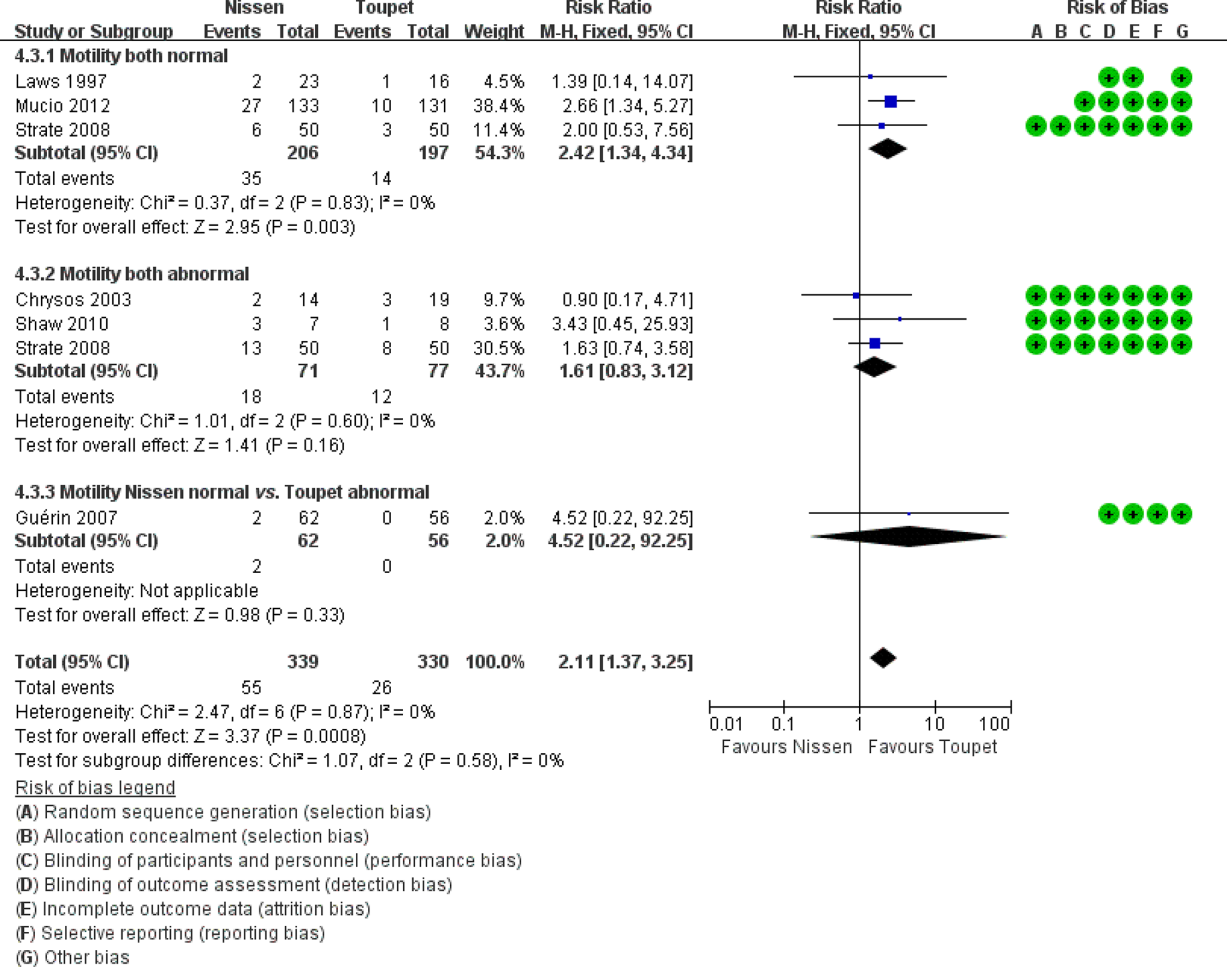
Subgroup analysis of dysphagia according to esophageal motility.

**Fig 4:**
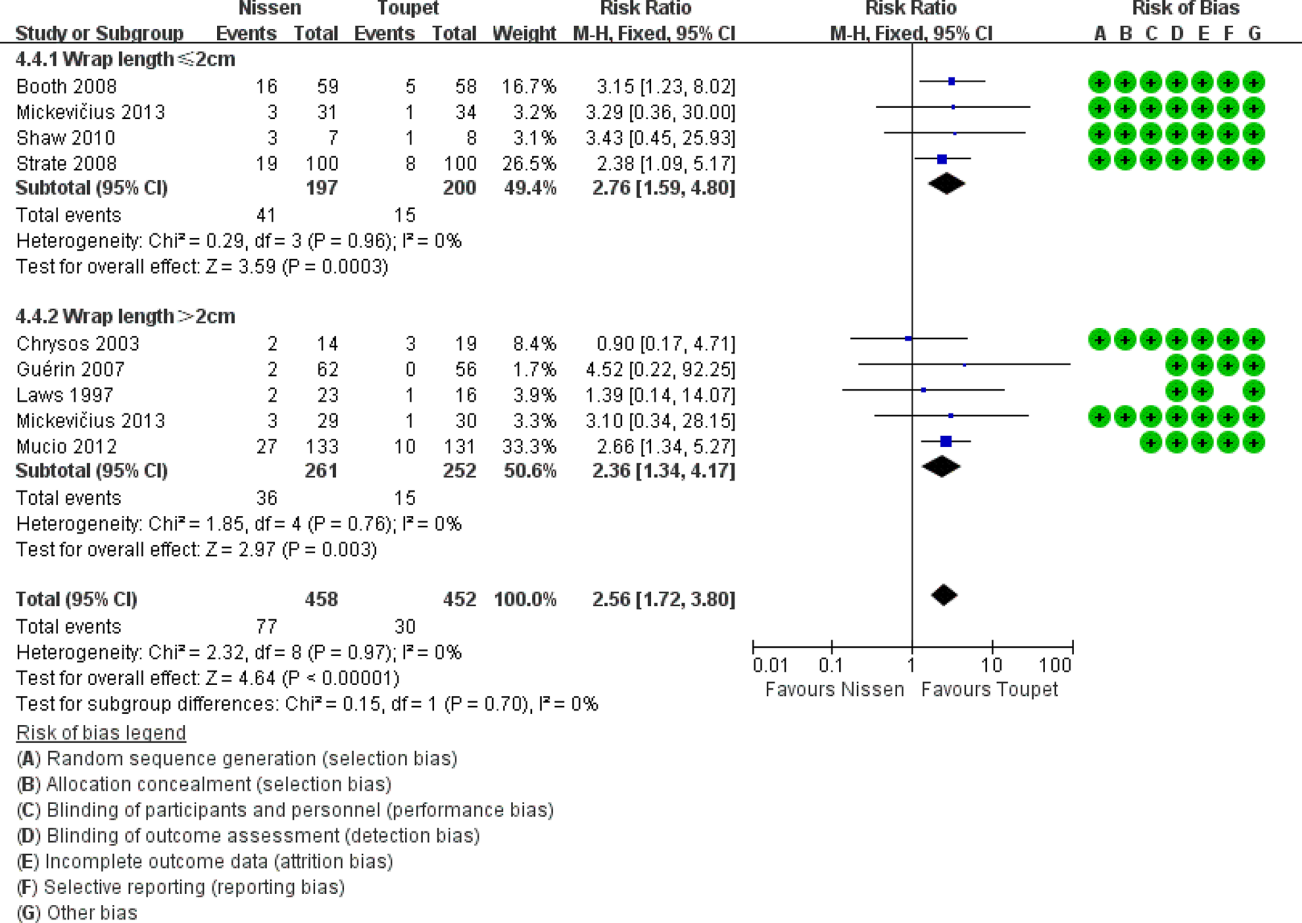
Subgroup analysis of dysphagia according to wrap length.

Apart from fundoplication type, EM and wrap length are two important factors for postoperative dysphagia; therefore, a subgroup analysis was performed in this area (Figs 3 and 4). In preoperative normal EM subgroups, postoperative dysphagia occurred more commonly following LTF compared with LNF (LNF 17% vs. LTF 7.11%, RR 2.42, 95% CI [1.34, 4.34], p = 0.003). In the subgroups of LTF with normal EM or abnormal EM, the prevalence of postoperative dysphagia were both showed lower than that in the subgroup of LNF with normal EM, even if there were no significant differences. In the subgroup analysis of patients with a wrap length ≤2 cm, LNF was associated with a significantly higher incidence of postoperative dysphagia. However, in the subgroup analysis of patients with a wrap length >2 cm, this difference was not found to be statistically significant (subgroup 1: LNF 20.81% vs. LTF 7.50%, RR 2.76, 95% CI [1.59, 4.80], p = 0.0003; subgroup 2: LNF 7.03% vs. LTF 4.13%, RR 1.79, 95% CI [0.64, 5.03], p = 0.27).

#### Gas-bloat syndrome or gas-related symptoms

The symptoms of gas-bloat syndrome include bloating, inability to belch, postprandial fullness, flatulence, and epigastric pain. Five trials [7, 12, 20–21, 24] assessed the outcome of gas-related symptoms (S6 Fig). The overall prevalence of gas-related symptoms was significantly higher after LNF vs. LTF (31.19% vs. 23.91%, RR 1.31, 95% CI [1.05, 1.65], p = 0.02). Inability to belch occurred in 33 of 221 (14.93%) patients following LNF and 18 of 214 (8.41%) patients following LTF, respectively. Therefore, there was a higher incidence of inability to belch for patients following LNF (RR 1.79, 95% CI [1.06, 3.03], p = 0.03) (S7 Fig). In addition, Booth et al. [21] reported that 18.64%/10.34% suffered from gas-bloat symptoms, 62.71%/63.79% had postprandial fullness, 74.58%/67.24% complained of flatulence, and 25.42%/31.03% experienced epigastric pain after both LNF and LTF.

#### Reappearance of GERD symptoms

**Fig 5:**
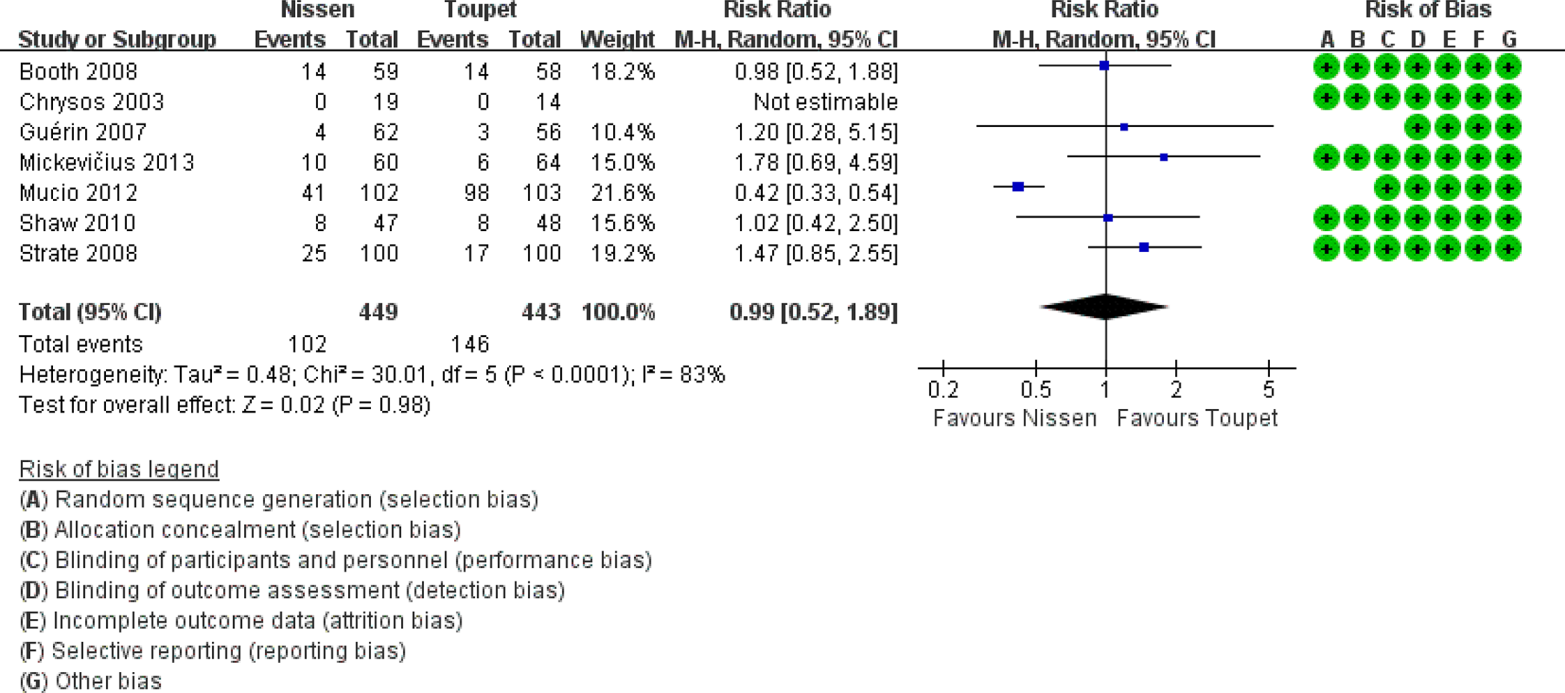
Rates of recurrence of GERD after LNF and LTF.

The postoperative reappearance of typical GERD symptomsincludes heartburn, regurgitation, chest pain. Heartburn is the most typical symptom of GERD and this symptom was considered to indicate recurrence. Reappearance of GERD symptoms was reported in seven trials [7, 12, 15, 20–21, 24–25], including 449 patients following LNF and 443 patients following LTF (Fig 5). The overall recurrence rate was 22.72% (102/449) after LNF and 32.96% (146/443) after LTF (RR 0.99, 95% CI [0.52, 1.89], p = 0.59). Subjective reflux control was similar for complete and partial fundoplication. Of the included patients, 13.01% suffered from heartburn after LF (LNF 14.00% vs. LTF 11.98%, p = 0.45), 6.57% had regurgitation (LNF vs. LTF: 3.41% vs. 10.42%, p = 0.54), and 6.72% complained of chest pain (LNF vs. LTF: 10.71% vs. 2.34%, p = 0.01).

#### Requirement for antireflux medication and reoperation

The need for antireflux medication was described in two trials [7, 25] and reoperation was described in four trials [15–16, 24–25] (S8 Fig and S9 Fig). A total of 14 of 209 patients after LNF and 13 of 112 patients after LTF required medication due to severe reflux after LF, but this difference was not found to be statistically significant (LNF vs. LTF: 13.08% vs. 11.61%, RR = 1.13, p = 0.74). Redo fundoplications for GERD recurrence were similar for complete and partial fundoplication (LNF vs. LTF: 4.74% vs. 6.54%, p = 0.77) [15–16, 25]. However, 11 patients [16, 24–25] in the LNF group suffered from severe postoperative dysphagia and underwent reoperation, whereas there were no patients with severe dysphagia in patients undergoing reoperation in the LTF group (S10 Fig).

### Objective evaluations

#### LES resting pressure

**Fig 6:**
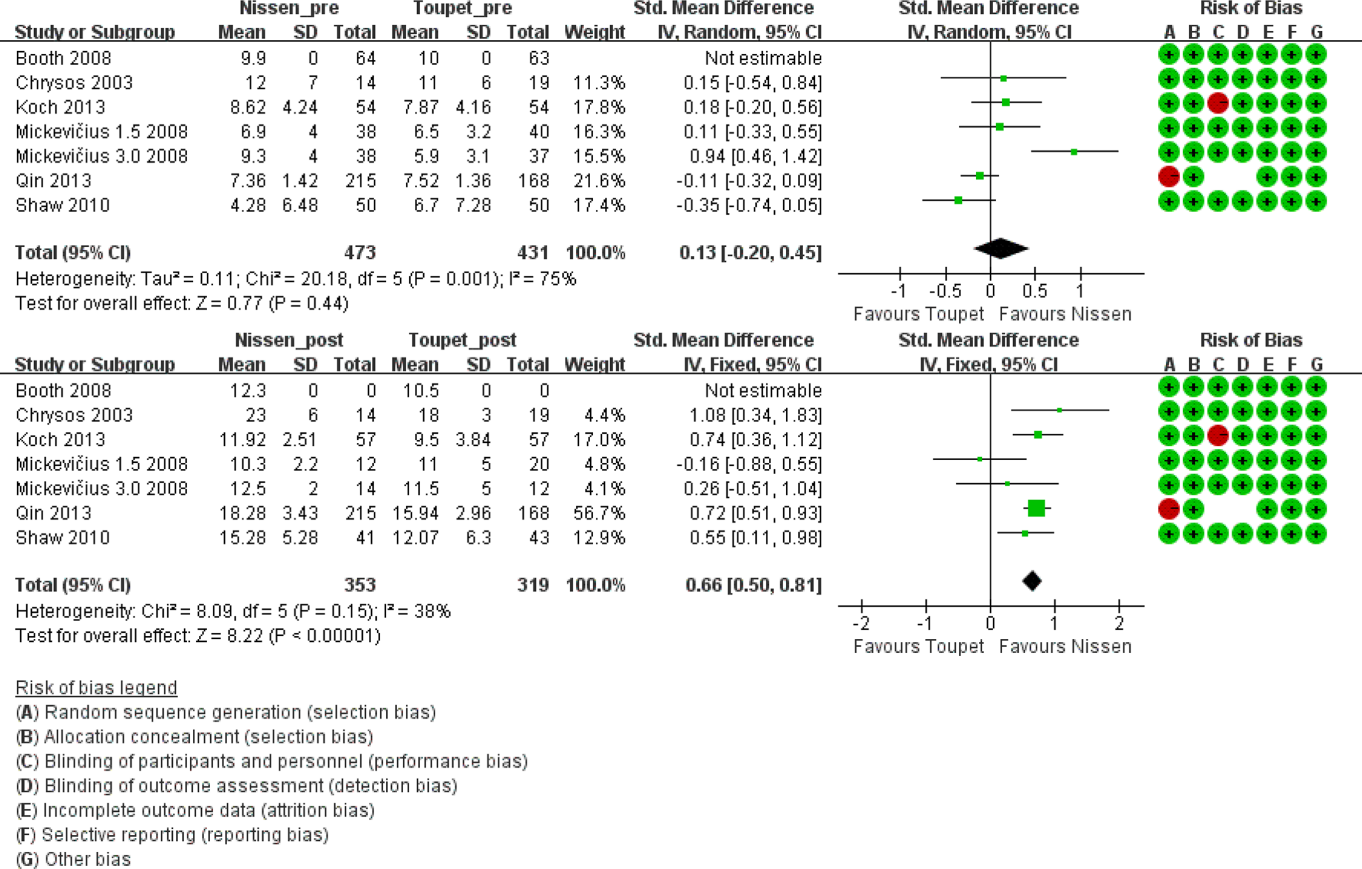
LES resting pressure before and after LNF and LTF.

Six trials [6, 12, 16, 17, 21, 25] measured LES resting pressure before and after LF. Preoperative average LES pressure ranged from 4.28 to 12 mm Hg in the LNF group and from 5.9 to 11 mm Hg in the LTF group. A significant improvement in LES pressure was achieved in both groups (10.3 to 23 mm Hg after LNF and 9.5 to 18 mm Hg after LTF). Preoperative LES pressure was approximate between groups. However, postoperative LES pressure increased significantly in the LNF group compared with the LTF group (Fig 6).

#### DeMeester score based on gastric monitoring of 24-pH

**Fig 7:**
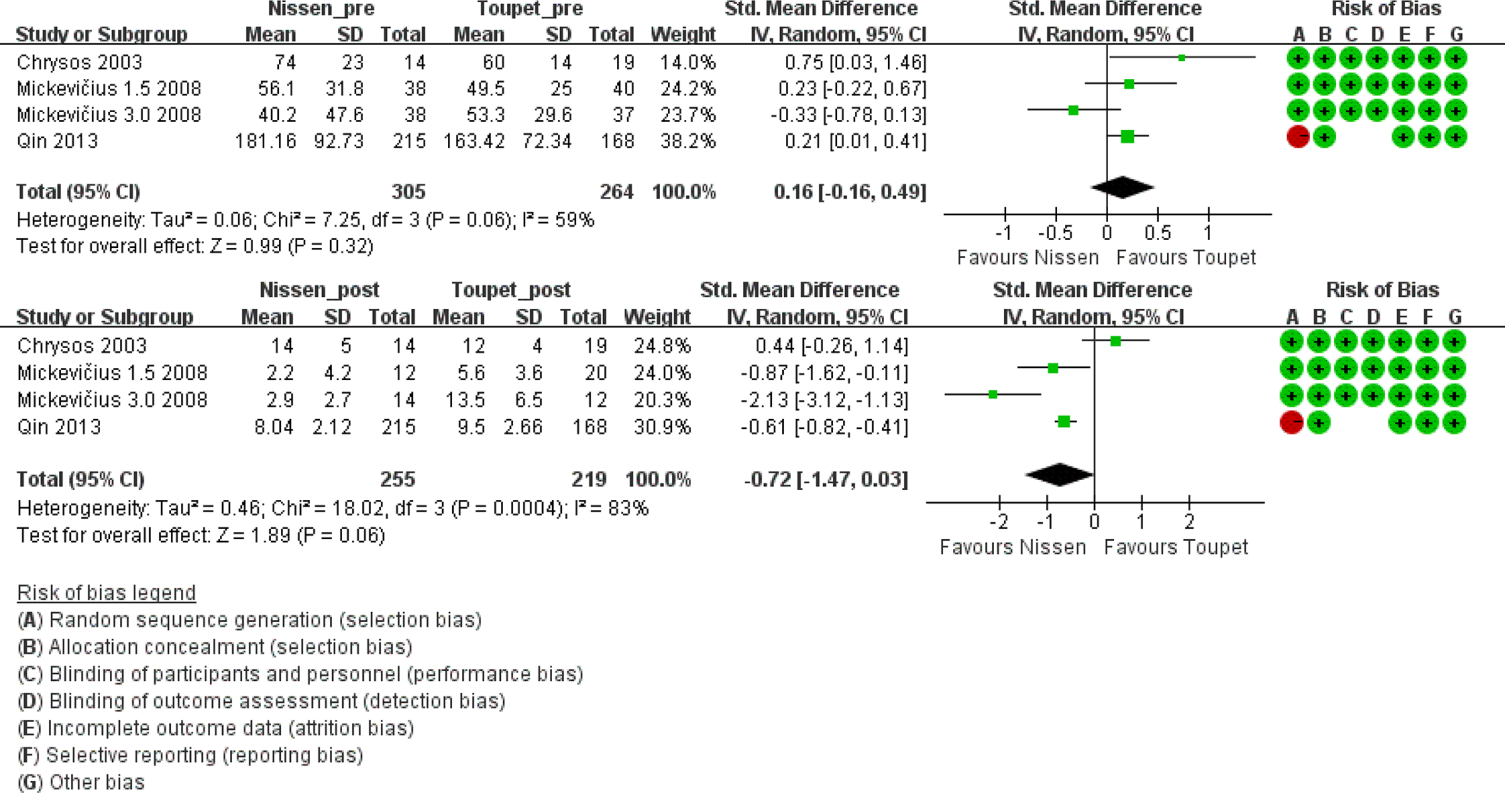
DeMeester score before and after LNF and LTF.

Three trials [6, 12, 17] performed 24-hour pH monitoring and provided both pre- and postoperative DeMeester scores. Although the preoperative score was not significantly different between groups, the postoperative DeMeester score was lower after LNF (pre-LNF vs. pre-LTF, standard mean difference 0.16, 95% CI [-0.16, 0.49], p = 0.32; post-LNF vs. post-LTF, standard mean difference -0.72, 95% CI [-1.47, 0.03], p = 0.06). However, the mean scores after both LNF and LTF were normal (i.e. <14.7) (Fig 7).

## Discussion

In our meta-analysis, we included 13 RCTs that reported outcome and postoperative complications of 1564 patients undergoing LNF or LTF. We found that LTF was equally effective in improving quality of life and controlling reflux compared to LNF, but had a lower incidence of postoperative dysphagia and other gas-related symptoms.

Subjective outcomes are accurate and important to evaluate the efficacy of LF for GERD. We demonstrated that both LNF and LTF significantly improve patients’ quality of life while providing optimal control of GERD symptoms. While the efficacy of laparoscopic antireflux surgery has been confirmed by our analysis, adverse effects such as dysphagia, gas-bloat syndrome, and others remain major postoperative complications. The prevalence of dysphagia after LTF was found to be significantly lower than after LNF. Anatomic factors including a tight wrap, distal migration of the wrap over the stomach, migration of the wrap into the mediastinum due to a shortened esophagus, or tight approximation of the crura had been initially implicated in the pathogenesis of postoperative dysphagia [12]. Modifications of total fundoplication, such as partial fundoplication or performing a loose fundoplication are now in common practice. Postoperative dysphagia was reported to occur in 20% to 40% of patients in previous studies [3, 4, 9, 26]. However, in the current analysis, the prevalence was only 12.56% and 4.84% after LNF and LTF, respectively. This phenomenon may be due to the fact that more trials with long-term follow-up were included in our analysis, with improving results as the follow-up period continued.

In the patients with preoperative esophageal dysmotility, dysphagia due to the restriction of LES opening during swallowing, caused by distortion of the distal esophagus at the wrap segment, is generally worse. Inadequate esophageal peristalsis cannot overcome this obstacle, and the emptying of the bolus into the stomach will be blocked [27, 28]. Based on this concept, it has been advocated that there is no need to conduct a Toupet procedure over a full wrap in these patients [29, 30]. Our subgroup analysis, which demonstrated a higher prevalence in both the abnormal EM subgroup compared with the normal subgroup, is consistent with this concept. However, the results in the three subgroups altogether favored LTF, even though it is not significantly different from subgroups 2 and 3. Thus, we still recommend partial fundoplication. It is the surgical approach that affects the prevalence of postoperative dysphagia, instead of preoperative esophageal motility.

It should be noted that dysphagia and preoperative esophageal dysmotility was not accurately defined in most included studies. To evaluate postoperative dysphagia, a subjective questionnaire was used to determine whether they had dysphagia or not during follow-up by Laws et al. [19]. Grading of dysphagia was done by applying Likert scale in the postoperative clinical assessment conducted by Mickevičius et al. [7]. To identify preoperative esophageal dysmotility, dynamic esophageal peristalsis amplitude at the distal esophagus or percentage of effective primary peristalsis during several swallows was most widely used. However, we found that the evaluation criterions were of great variation among studies. For example, Mucio et al. and Strate et al. determined esophageal dysmotility as less than 70% and 60% effective peristalsis, respectively [15][24]. This factor might affect the ability to draw meaningful conclusions about choosing the adequate surgery approach, in spite of the negligible heterogeneity of analysis concerning the postoperative dysphagia with or without preoperative impaired EM. Chicago classification is the newest and the most comprehensive criteria of diagnosing EM disorders to date, which was defined by utilizing high resolution esophageal pressure topography incorporating the combination of high resolution manometry instead of traditional esophageal manometry [31]. Unfortunately, none of included studies in our meta-analysis used Chicago classification to evaluate esophageal dysmotility. For accurate definitions of EM disorders, Chicago classification is required to be used in further studies.

The cause of the gas-bloat syndrome has been attributed to several complex anatomic and functional factors, including vagus nerve injury, slippage, dislocation or disruption of the wrap, defective LES relaxation, preexisting gastric motility disorders, or even to a completely different mechanism of belching in postfundoplication patients [12]. A recent study demonstrated that belching pattern is altered by LNF, by reducing gastric belching (air venting from the stomach) and increasing supragastric belching (no air venting from the stomach) [32]. In our study, LTF was superior in terms of gas-bloat syndrome. A tendency for fewer gas bloat symptoms after LTF in the short- and long-term follow up period has also been reported in prior studies [33, 34]. Taking into consideration the technical details of constructing a partial posterior fundic wrap, LTF appears to be more physiologic than the LNF [35].

In this meta-analysis, reappearance of GERD symptoms was not found to correlate with GERD recurrence. GERD symptoms may result from acid reflux, esophageal hypersensitivity, sustained esophageal contractions or abnormal tissue resistance [36]. Esophageal hypersensitivity may be an independent phenomenon or may overlap with GERD. It describes a condition in which an esophageal stimulus induces GERD symptoms but without any esophageal injury. In other words, patients with esophageal hypersensitivity have a lower threshold for the perception of physiologically nonpainful stimuli [37]. In our study, reappearance of GERD symptoms occurred in 55.2% (449/814) patients following LNF and 59.1% (443/750) patients following LTF. However, the mean scores of DeMeester after both LNF and LTF were normal (i.e. <14.7), which was not accordance with occurrence of postoperative GERD symptoms. Therefore, several recent publications held the view that the recurrence of GERD should be identified by pH studies instead of simply subjective symptoms [38, 39]. Actually, only 26% of symptomatic patients in these studies had abnormal pH studies results. There was no need for these patients to return to PPI treatment. The indication for postoperative medication or re-operations should rely on objective results. As the indication for therapy in GERD depends mainly on clinical symptoms [24], LTF has the same success as LNF in the terms of recurrence. Previous studies reported possible higher recurrence rates of GERD after partial posterior fundoplication [40, 41]. It has been shown that the technique of LTF, consisting of a more than 300° wrap without dissection of the greater curvature, creates an antireflux barrier which is as effective as in LNF [40, 42–43]. In addition, the requirement for postoperative antireflux medications or reoperation due to severe symptoms can also help to determine which approach has a more satisfactory result. Because LNF is a total wrap, postoperative LES pressure was higher compared with LTF, whereas this difference was not apparent in the preoperative period. Although the DeMeester score was lower after LNF, the mean postoperative score was normal after both LNF and LTF, which suggested that reflux can be controlled by LTF to normal level. Meanwhile, we observed a higher prevalence of postoperative adverse results after LNF, corresponding with higher LES pressure. LTF tends to have less outflow resistance as suggested by a lower LES pressure and greater degree of LES relaxation, resulting in postoperative improvement of esophageal peristalsis and esophageal emptying by constructing a physiologic antireflux barrier, while LNF may result in a decreased ability to clear the esophagus [40, 44]. In our current analysis, however, those included studies didn’t provide objective long-term data beyond 5 years. The data of postoperative asymptomatic patients who refused the physiological invasive examinations or lost to follow-up was unavailable [25]. The LTF has always been criticized as having decreased durability in the long-term as several large prospective non-randomized and retrospective studies with long-term follow-up suggested poorer long-term reflux control after LTF [45, 46]. In the study conducted by Kamolz et al., LES pressure decreased over time following LTF but increased over time following LNF. But DeMeester scores were both decreased after 5 years following LNF and LTF [47]. Uncertainty exists over what level of LES pressure can prevent reflux while still avoiding dysphagia. Based on our experience, a postoperative increase in LES pressure of 2 to 5 mmHg below the normal value (10–12 mm Hg) is sufficient to control reflux. Moreover, even though the increase in LES pressure was significantly less after LTF than after LNF, the increase seemed sufficient to prevent the reflux of gastric contents. Results of the present study also support the view that LTF can control reflux symptoms for GERD.

Finally, we concluded that LTF may be the superior surgical approach for GERD with a lower morbidity rate and equal effectiveness compared with LNF.

## Supporting Information

S1 Table PRISMA 2009 Checklist for systemic review and meta-analysis. (PDF)

S1 Fig PRISMA 2009 Flow Diagram. (TIF)

S2 Fig Forest-plot showing the operative time of LNF and LTF. (PNG)

S3 Fig Perioperative complications after LNF and LTF. (PNG)

S4 Fig Postoperative satisfaction after LNF and LTF. (PNG)

S5 Fig Rates of moderate to severe dysphagia after LNF and LTF. (PNG)

S6 Fig Rates of gas bloat syndrome after LNF and LTF. (PNG)

S7 Fig Rates of inability to belch after LNF and LTF. (PNG)

S8 Fig Rates of medication application after LNF and LTF. (PNG)

S9 Fig Rates of re-operation due to GERD recurrence after LNF and LTF. (PNG)

S10 Fig Rates of re-operation due to severe dysphagia after LNF and LTF. (PNG)
