# Supplementary figures and images for: A Meta-Analysis of Randomized Controlled Trials to Compare Long-Term Outcomes of Nissen and Toupet Fundoplication for Gastroesophageal Reflux Disease
Source: PLoS One. 2015 Jun 29;10(6):e0127627. doi: 10.1371/journal.pone.0127627 (PMC4484805; doi:10.1371/journal.pone.0127627)

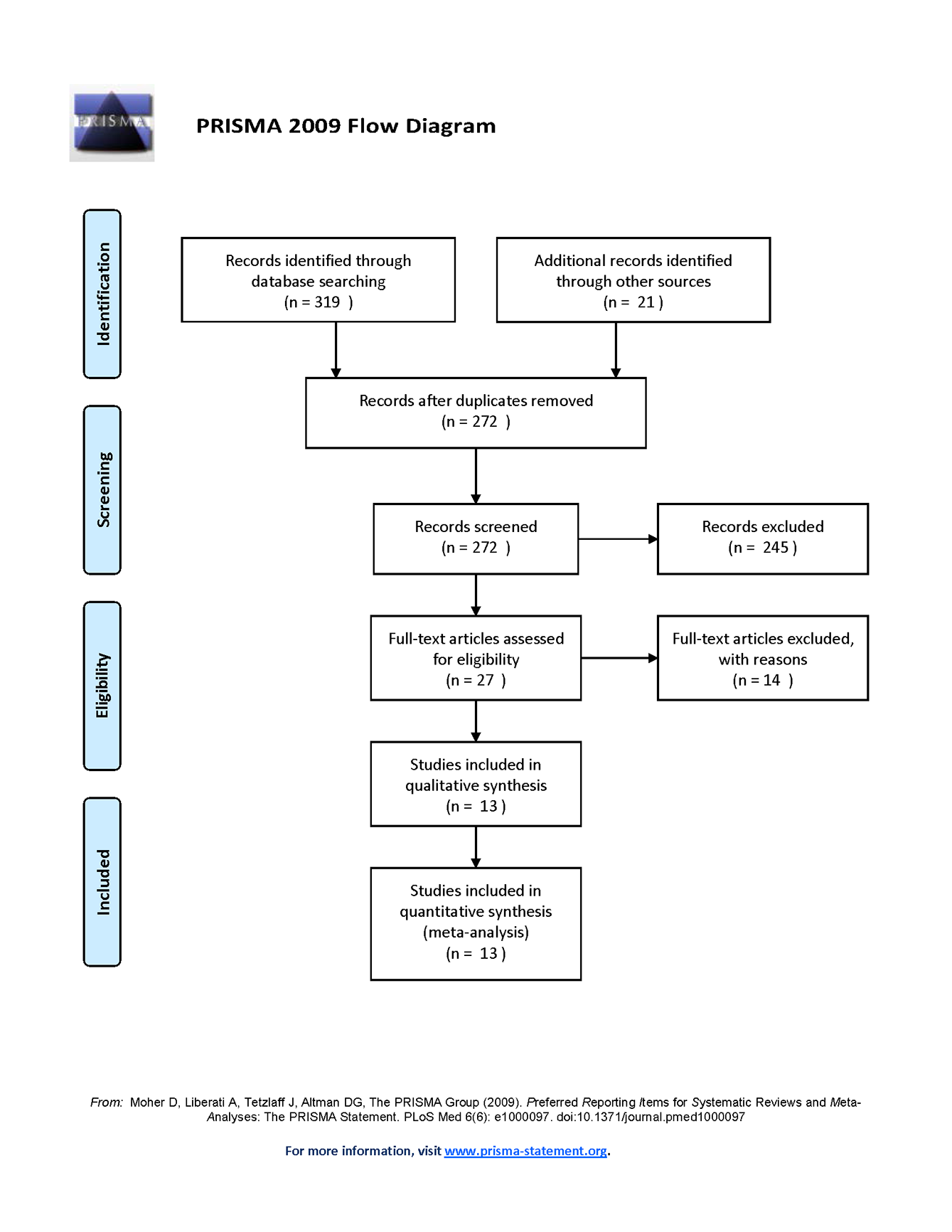

Supplement: S1 Fig — (TIF) [file pone.0127627.s002.tif]

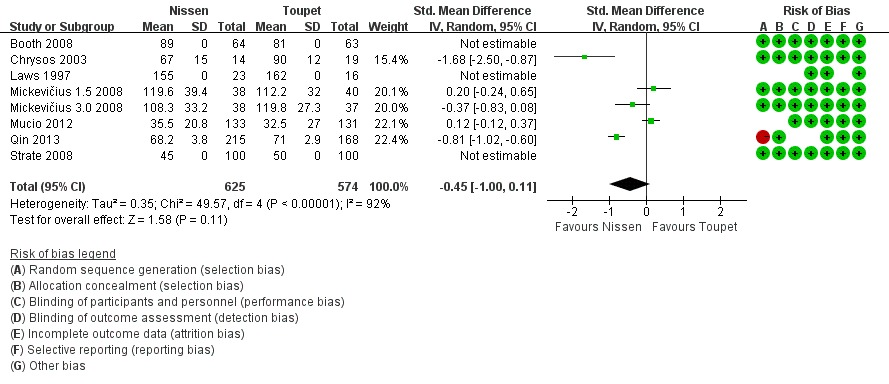

Supplement: S2 Fig — (PNG) [file pone.0127627.s003.png]

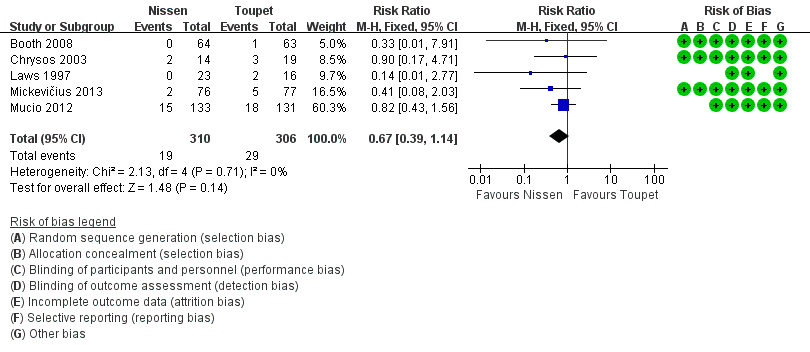

Supplement: S3 Fig — (PNG) [file pone.0127627.s004.png]

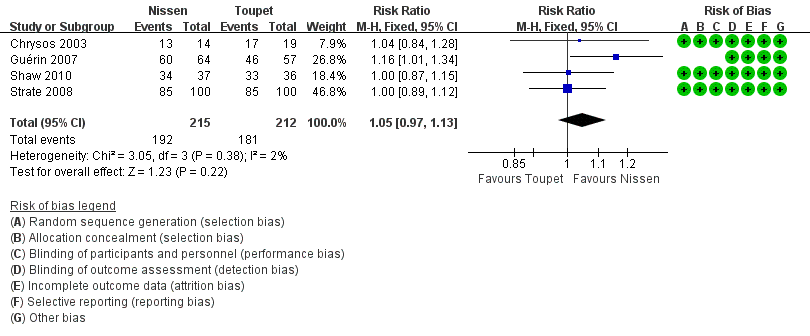

Supplement: S4 Fig — (PNG) [file pone.0127627.s005.png]

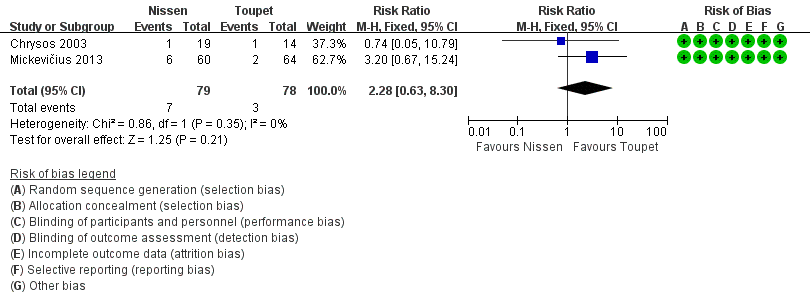

Supplement: S5 Fig — (PNG) [file pone.0127627.s006.png]

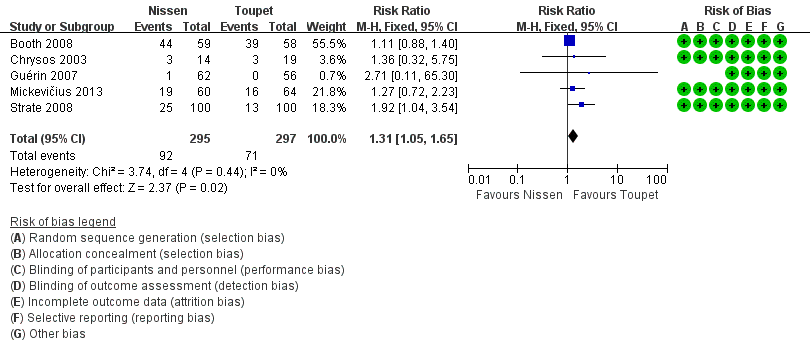

Supplement: S6 Fig — (PNG) [file pone.0127627.s007.png]

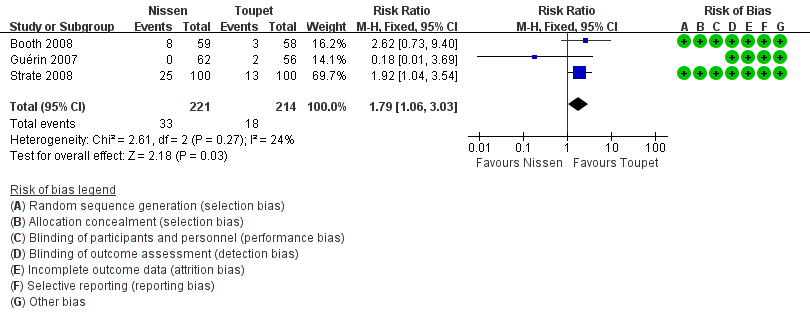

Supplement: S7 Fig — (PNG) [file pone.0127627.s008.png]

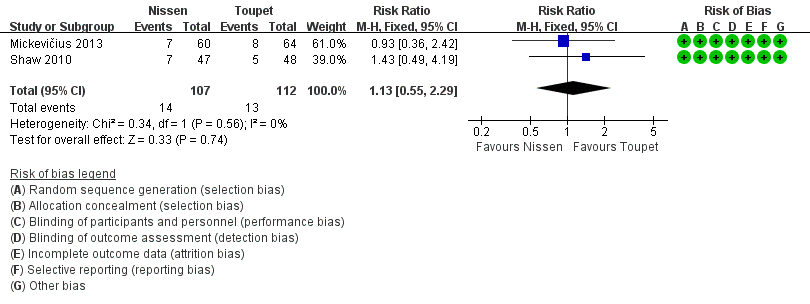

Supplement: S8 Fig — (PNG) [file pone.0127627.s009.png]

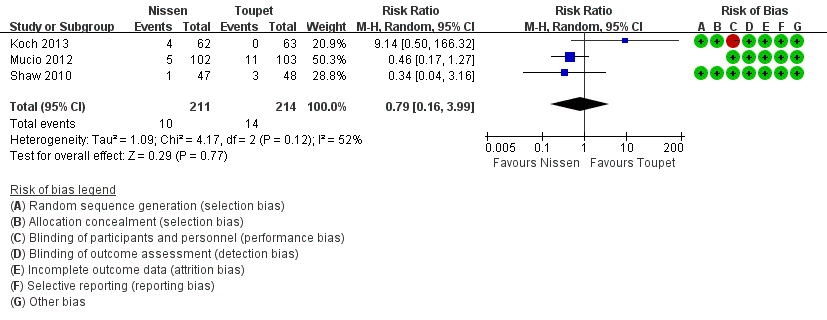

Supplement: S9 Fig — (PNG) [file pone.0127627.s010.png]

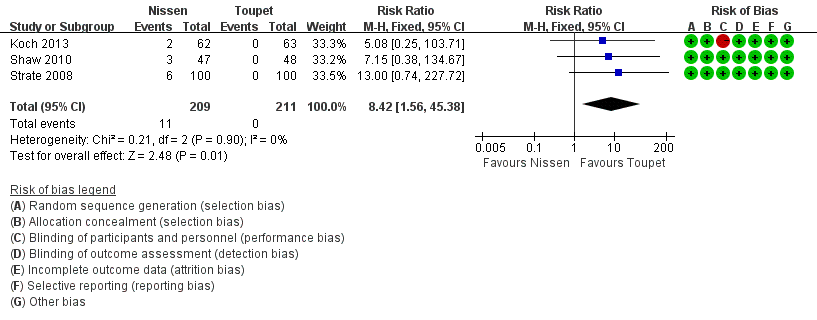

Supplement: S10 Fig — (PNG) [file pone.0127627.s011.png]
